# Computational modeling of peripheral pain: a commentary

**DOI:** 10.1186/s12938-015-0049-x

**Published:** 2015-06-11

**Authors:** Erick J Argüello, Ricardo J Silva, Mónica K Huerta, René S Avila

**Affiliations:** Laboratorio “C” at Universidad Simón Bolívar, Caracas, Venezuela; Secretaría de Educación Superior, Ciencia, Tecnología e Innovación (SENESCYT), Guayaquil, Ecuador; Programa Promeinfo, Universidad de Guayaquil, Guayaquil, Ecuador; Universidad Politécnica Salesiana (UPS), Cuenca, Ecuador; Grupo de Redes y Telemática Aplicada at Universidad Simón Bolívar, Caracas, Venezuela

**Keywords:** Computational pain model, Noxious processing, Gate control theory, Artificial neural network, Neuron models

## Abstract

This commentary is intended to find possible explanations for the low impact of computational modeling on pain research. We discuss the main strategies that have been used in building computational models for the study of pain. The analysis suggests that traditional models lack biological plausibility at some levels, they do not provide clinically relevant results, and they cannot capture the stochastic character of neural dynamics. On this basis, we provide some suggestions that may be useful in building computational models of pain with a wider range of applications.

## Background

New approaches to address the multidimensional character of pain include the utilization of mathematical and computational models, whose main advantages lie on the noninvasive nature of the method (which is particularly important, from an ethical point of view, in performing studies with chronic pain patients) and on their capabilities to predict previously unnoticed behaviors [1, 2]. Specifically regarding peripheral pain (i.e. the kind of pain than can be elicited by activating primary afferent neurons), some models are able to capture the cellular and molecular basis of noxious stimuli processing [3–6], while others use artificial neural networks (ANN) under the assumption that only these parallel-distributed processors can mimic the computational capabilities owned by the nervous system [7–10]. However, a very few such studies have been published over the last 5 years [10–12], even though the computational power available to us has increased. Whether this is due to the difficulties of modeling a subjective experience, or the limited use of computational models in pain research, it is hard to say. Still, possible explanations for the latter can be provided below.

This commentary discusses the main strategies that have been used for the computational modeling of peripheral pain, which includes all those studies able to provide a quantitative description of the neurobiological processes that precede or accompany the pain experience, including the transduction, transmission and modulation of cutaneous noxious stimuli. Interestingly, such studies represent a very low percentage of the total of cites yielded by using “computational”, “model” and “pain” as keywords (a search on ScienceDirect yields 13.000 cites, but no more than few tens meet the aforementioned requirements).

## Quantitative modeling of peripheral pain

Pain is a subjective experience and, as such, it cannot be the object of empirical study, let alone described in quantitative terms. Nevertheless, it involves the activation of specific supraspinal structures, such as primary and secondary somatosensory, insular, anterior cingulate, and prefrontal cortices (S1, S2, IC, ACC, PFC) and the thalamus (Th), as revealed by a meta-analysis of human data from different imaging studies [13]. These structures are activated in turn by nerve impulses transmitted from the dorsal horn (DH) by projection neurons, once the peripheral noxious stimuli are processed at spinal level. Unlike perceptual experiences, this kind of events can be experimentally measured as well as translated into quantitative terms to build a mathematical model. Thus, most (if not all) computational models of pain are not able to predict the perceptual dynamics of such experience, but rather a series of conditions, both external (e.g. the incoming stimuli) and internal (e.g. the interactions between the parts of the model), that could lead to its manifestation.

With regard to the architecture defined by the linkages between their fundamental processing units, computational models of peripheral pain may fall into one of two categories: one including the models based on ANN and one including all others.

### ANN-based models of peripheral pain

Inspired by biological neural networks, the ANN are parallel-distributed processors composed of a very large number of elementary units and they have been developed to mimic the processing capabilities owned by the human brain, which can outperform those exhibited by conventional computers when a complex perceptual problem needs to be solved (such as recognizing a familiar face in a crowd) [14]. Hence it is reasonable to think that some features of pain (a perceptual phenomenon) can be captured and quantitatively described by ANN. A pioneering study of this type was carried out by Minamitami and Hagita [7], who proposed a computational model able to provide temporal firing patterns of supraspinal neural units, including the S1 and S2 cortical cells, as a function of single pulse and repetitive stimulation. Haeri et al. [8] modeled steady-state and dynamic behavior of noxious stimuli processing by using a multilayer perceptron (MLP) (one hidden layer) and a recurrent neural network (RNN), respectively. Both models were trained after extracting the proper features from input/output patterns associated with acute and chronic pain. A more recent study performed by de Sousa and de Jesus Torres [10] introduced an ANN-based model of peripheral pain capable of being implemented on a field programmable gate array (FPGA). For this purpose, a novel method for adjusting both the inflexion points and the slope of the activation function of neural units was also described. In general, the results yielded by this kind of models are in good agreement with experimental and clinical observations, and recent efforts to allow hardware implementations may contribute to develop future portable applications in the medical field.

### Models not based on ANN

The majority of computational models of peripheral pain belonging this category use the neural circuit proposed in the gate control theory (GCT) [15], either to define the architecture of the model [3, 11], or as the equation system [4, 6, 16] brilliantly distilled by Britton and Skevington [17], only a few decades after the theory was published. On the other hand, a much reduced number of studies provide alternative representations. Farajidavar et al. [5], for example, explained some of the underlying mechanisms of a relatively uncommon type of wind-up that can be elicited by stimulation of Aβ fibers in hyperalgesic states, by using transfer functions in a block diagram. In a subsequent study [18], a single 3-to-1 network was used to propose that long-term changes in synaptic efficacy that depend on synchronous firing between pre- and post-synaptic cells may be responsible for the synaptic potentiation leading to wind-up, whereas short-term synaptic changes could explain why wind-up only occurs within a narrow range of frequencies.

Several attempts have been made to exploit (and, perhaps, to balance) the extremely simple depiction of the gate mechanism introduced by Melzack and Wall. The groups of Agi and Xu, for example, addressed from a multi-level perspective the transduction, transmission and modulation of the nerve impulses evoked by noxious stimuli, either by including morphological and functional characteristics exhibited by different subpopulations of afferent and DH neurons [3], or by taking into account the thermo-mechanical response of the skin tissue [6]. Prince et al. [16] implemented 2, 10, 50 and 200 copies of the GCT circuit in order to observe how the descending inhibitory control varied as a function of the number of elements involved in the modulation of sensory information after calculating the sum of all outputs, taken at the T cell of each copy. As the computational power available has increased, it is now possible to enlarge elementary networks by incorporating more components and details, which in turn, adds biological plausibility to computational models of pain. But, if this is so, why computational modeling tends not to have much impact in the field of pain?

## Biological plausibility is observed at several, but not all, levels of the model

In general, ANN-based models of peripheral pain have provided results in good agreement with experimental and clinical observations, and recently, some efforts have been made to allow hardware implementations [10]. In addition, the architecture of these models can be as complex as that exhibited by biological neural networks involved in processing of noxious information. However, the main building block is too simple to capture the intrinsic properties of different neuronal subpopulations, an aspect that has become more relevant in the light of new pain theories that imply some degree of peripheral specificity [19, 20]. Synaptic weights are typically obtained by using training algorithms, such as the backpropagation algorithm [14], which reverts the flow of nerve impulses (from the axon terminal to the soma and then to the dendrites) and, therefore, lacks biological plausibility. Numeric values are commonly normalized to fit the implementations, so they may lose their physiological meaning, and by definition, ANN-based models are only capable of mapping a finite number of inputs (stimulation patterns) to their corresponding (desired) outputs, so results tend not to be good as new data are provided.

To aid in understanding the neural basis of noxious stimuli processing, a significant amount of studies have focused on describing in detail the dynamics of the action potential and analyzing the contribution of multiple aspects (e.g. morphology, intra- and extracellular ion concentrations) in its generation and transmission. Still, these aspects have only been modeled within a network composed by a very low number of elements [3, 6], or in isolation [21–23], probably due to the common view that biophysically accurate neuron models, such as the model of Hodgkin and Huxley (HH) [24], are computationally prohibitive for solving problems that involve a large number of units. This tendency may seriously limit the use of computational models for the study of pain, since the neuron connectivity, especially at central level, has been overlooked.

## Do simulations yield clinically relevant results?

Although pain often acts as a warning signal which alerts us about injury and disease, it may also occur as an amplified response to stimulation, either harmful or innocuous, or even spontaneously (i.e. in the absence of noxious stimuli). Under these circumstances, the pain experience loses its protective function and becomes a debilitating condition, often developed by a series of maladaptive plastic changes of the somatosensory nervous system [25]. In clinical practice, chronic pain and neuropathies are far more important than nociceptive pain, so the majority of computational models, which are capable of describing only some mechanisms related to the latter, are unable to predict clinically relevant aspects.

In addition to their predictive capabilities, computational models allow us to generate theoretical findings with some implications for pain management. The results yielded by a computational model of thermal pain [6], suggest that existing heat therapies for burn treatment can be optimized by predicting the damage that have been accumulated in the skin tissue during heating. By implementing a computational model of wind-up, Farajidavar et al. [18] proposed that transcutaneous electrical stimulation of afferent fibers (TENS) with the appropriate spike timings may decrease the synaptic efficacy in nociceptive transmission and, therefore, contribute to pain relief. However, if all those hypotheses remain untested, then the results generated from the implementations become practically useless for medical purposes.

## Stochastic character of neural dynamics have been neglected

The opening and closing of ionic channels are probabilistic events, and there is spontaneous release of neurotransmitter that elicits small depolarizations and hyperpolarizations at random. By contrast, most, if not all, computational models of peripheral pain are essentially deterministic and, therefore, unable to account for the role of several (or chaotic) random phenomena, such as the spontaneous neural activity that tends to be weaker than the activity induced by actual stimulation under normal circumstances, but increases abnormally after deafferentation [25].

## Future directions in computational modeling of pain

Now that possible explanations for the limited use of computational modeling in pain research have been provided, the following section outlines what we consider to be useful in addressing those issues.

*Achieving true interdisciplinarity* It implies not only to incorporate different fields of expertise in building computational models of pain, but also to collaborate in testing theoretical findings yielded by their implementation. If the hypotheses are true, then those models may prove to be useful for assessing and/or improving the efficacy of well-established strategies for pain relief, as well as for developing innovative approaches. Otherwise, opportunities for future research may emerge either by reformulating current models or by generating new alternatives based on recent evidence. Of course, more experimentalists must be willing to share their data with modelers, who in turn must be willing to make their codes available. Likewise, governmental entities and university faculties also need to play a more active role, not only by funding the best graduates with competitive interdisciplinary fellowships, but also by changing the administrative and cultural framework that make these approaches so hard to accomplish, especially for those who choose this path early in their careers.

**Figure 1:**
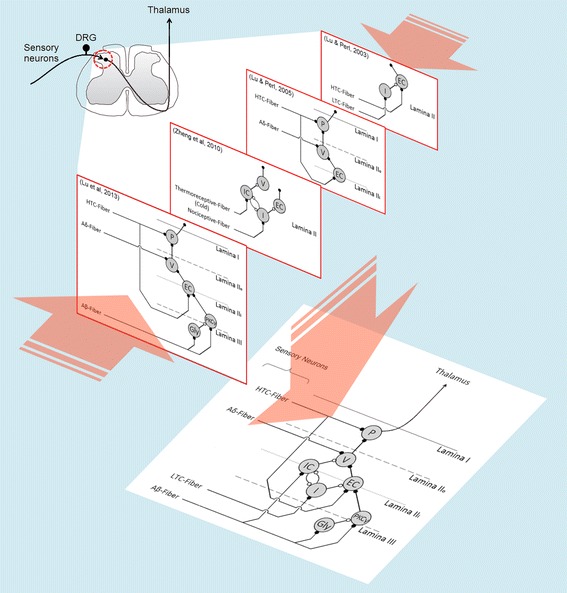
Experimental results yielded by different studies can be combined to build an alternative representation of the pain circuitry. Unidirectional synaptic connections among superficial dorsal horn neurons have been reported by Lu and coworkers (*red bordered panels*) [26–29]. Each study per se only provides a piece of information on neuron connectivity at that region, but these pieces can be put together, by identifying the elements they have in common, to build a network reflecting some of the interactions that actually contribute to noxious information processing (*bottom panel*). *EC* excitatory central cell, *Gly* glycinergic neuron, *HTC* high-threshold C-fiber, *I* islet cell, *IC* inhibitory central cell, *LTC* low-threshold C-fiber, *P* projection neuron, *PKCγ* neuron expressing the γ-isoform of protein kinase C, *V* vertical cell.

*Incorporating recent neuroanatomical evidence* Current computational models of peripheral pain cannot provide an accurate (or, at least, more realistic) representation of the circuitry responsible for noxious stimuli processing, especially at spinal level. This is probably due to the neural substrate involved in pain experience is still poorly understood. On the other hand, a significant amount of neuroanatomical evidence has become available over the last two decades (e.g. [26–31]). Although each of those studies only provides a piece of information on neuron connectivity at the superficial DH, they have some elements in common, such as the lamina II excitatory central cells, which receive inhibitory input from islet cells [26, 29], as well as excitatory input from high-threshold unmyelinated fibers and neurons expressing the protein kinase C isoform [27, 28]. Thus, it would be possible to put the pieces together and build a network (as depicted in Figure 1) that may reflect some of the interactions that actually contribute to the noxious information processing. Computational models of pain reflecting neuroanatomical findings are likely to provide more insights regarding how pain circuits perform their computations and how structural changes may lead to abnormal pain sensations resulting from an improper processing, so such models should be able to include the newest experimental evidence.

*Getting the most of available simulation tools* Among the countless neuron models capable of producing a wide range of firing patterns, only a few [24, 32] have been used in modeling of peripheral pain and their utilization has been, in turn, very limited. It has been recently demonstrated [33] that, contrary to common view, the HH model is not computationally prohibitive, especially nowadays that the computational capabilities have significantly increased. It is now feasible to model large-scale networks composed of spiking neurons, given the increasing number of freely available, open source and documented simulation environments (for detailed review, see [34]), so the utilization of this type of neuron models should be continuously encouraged.

*Implementing stochastic (chaotic) neural models and networks* Wind-up is a kind of plasticity that has been successfully modeled at the cellular and molecular levels [4, 5], as well as at the level of ANN [8]. Nevertheless, most of the plastic changes involved in the development of pathological pain states (e.g. chronic pain) cannot be described by these computational models, probably because they cannot capture the stochastic character of neuron dynamics. In a preliminary survey, Picton et al. [2] proposed that computational models of chronic pain must be able to acknowledge the role of spontaneous neural activity and be self-organizing, and that chaotic neural networks may fulfill these requirements. A recent model developed by Böstrom et al. [12] was capable of reconciling apparently contradictory evidence related to the experience of phantom limb pain by including the spontaneous neural activity in the form of randomly occurring events. The model cannot account for the abnormal enhancement of spontaneous activity after deafferentation, but it can predict the maladaptive reorganization of the S1 cortex that occurs after amputation. Still, further work is needed to assess the suitability of chaotic models for computational modeling of pathological pain states.

The scarce number of studies on the computational modeling of peripheral pain may reflect not only an incomplete comprehension of the mechanisms involved in such experience, but also a mismanagement of the available resources. An example for the latter is provided as follows: the neuron model developed by Izhikevich is able to reproduce a wide range of firing patterns, many of which have been observed in DH neurons and whose implications for sensory processing need further investigation. However, this neuron model has been rarely used in computational modeling of pain (the model only was used in [18]) and its capabilities have been not fully exploited (it was used to represent one single tonic-firing neuron).

Ultimately, the views expressed above are not intended as a procedure to elaborate a computational model of peripheral pain able to account for all the experimental observations since, by definition, no model can do that. Instead, this comment only provides some suggestions that may be useful in building computational models of pain with a wider range of applications, including the predictive modeling of clinically relevant aspects.

## Abbreviations

ACC: anterior cingulated cortex
ANN: artificial neural networks
DH: dorsal horn
FPGA: field programmable gate array
GCT: gate control theory
HH: Hodgkin and Huxley
IC: insular cortex
MLP: multilayer perceptron
PFC: prefrontal cortex
RNN: recurrent neural network
S1: primary somatosensory cortex
S2: secondary somatosensory cortex
TENS: transcutaneous electrical nerve stimulation
Th: thalamus
